# Is sex by age interaction the missing factor in acute kidney injury epidemiology?

**DOI:** 10.1093/ndt/gfag064

**Published:** 2026-03-20

**Authors:** Francesca Cappadona, Emilio Venturelli, Marita Marengo, Stefania Prenna, Marco Fiorentino, Paolo Fabbrini, Erika Naso, Elisa Russo, Francesca Viazzi, Giuseppe Castellano, Vincenzo Cantaluppi, Pasquale Esposito

**Affiliations:** Unit of Nephrology, Dialysis and Transplantation, IRCCS Azienda Ospedaliera Metropolitana (IRCCS AOM), Policlinico San Martino, Genova, Italy; Department of Internal Medicine and Medical Specialties (DIMI), University of Genova, Genova, Italy; Nephrology and Dialysis Unit, ASLCN1, Cuneo, Italy; Nephrology and Kidney Transplantation Unit, Department of Translational Medicine (DIMET), University of Piemonte Orientale (UPO), AOU Maggiore della Carità, Novara, Italy; Nephrology, Dialysis and Transplantation Unit, Department of Precision and Regenerative Medicine and Ionian Area, University of Bari Aldo Moro, Bari, Italy; Nephrology and Dialysis Unit, ASST Nord Milano, Italy; Nephrology and Dialysis Unit, ASLCN1, Cuneo, Italy; Unit of Nephrology, Dialysis and Transplantation, IRCCS Azienda Ospedaliera Metropolitana (IRCCS AOM), Policlinico San Martino, Genova, Italy; Department of Internal Medicine and Medical Specialties (DIMI), University of Genova, Genova, Italy; Unit of Nephrology, Dialysis and Transplantation, IRCCS Azienda Ospedaliera Metropolitana (IRCCS AOM), Policlinico San Martino, Genova, Italy; Department of Internal Medicine and Medical Specialties (DIMI), University of Genova, Genova, Italy; Department of Nephrology, Dialysis and Renal Transplantation, Fondazione IRCCS Ca' Granda Ospedale Maggiore Policlinico, Milan, Italy; Nephrology and Kidney Transplantation Unit, Department of Translational Medicine (DIMET), University of Piemonte Orientale (UPO), AOU Maggiore della Carità, Novara, Italy; Unit of Nephrology, Dialysis and Transplantation, IRCCS Azienda Ospedaliera Metropolitana (IRCCS AOM), Policlinico San Martino, Genova, Italy; Department of Internal Medicine and Medical Specialties (DIMI), University of Genova, Genova, Italy

**Keywords:** acute kidney injury, age, epidemiology, mortality, sex

## Abstract

**Background and hypothesis:**

Acute kidney injury (AKI) is a multifactorial syndrome associated with increased morbidity and mortality during hospitalization and in the long-term follow-up. Emerging evidence suggests that biological sex and age may influence kidney damage and recovery; however, the specific role of these biological variables on AKI outcomes remains to be fully elucidated. This retrospective study aimed to explore the impact of sex and age on AKI epidemiology and outcomes in a large cohort of hospitalized adults.

**Methods:**

We analyzed clinical and administrative data from 87 087 adult patients admitted to two Italian university hospitals between 2016 and 2019. Excluding patients with stage 5 chronic kidney disease (CKD), we assessed AKI based on Kidney Diseases Improving Global Outcomes criteria using serum creatinine trends during hospitalization. Demographic characteristics, comorbidities, estimated glomerular filtration rate (eGFR), hospital stay, and outcomes were evaluated, with a focus on sex-based comparisons.

**Results:**

AKI occurred in 20.6% of patients, with a higher incidence in females (21.4%) than males (19.8%). Female patients were older and had lower eGFR at hospital admission, less incidence of diabetes and CKD, but a more frequent diagnosis of congestive heart failure. While stage 3 AKI was more common in males, undiagnosed AKI was more frequent in females with stage 1 AKI. Of note, age-stratified analysis revealed a dynamic sex-related risk: AKI incidence was higher in males in younger age groups, whereas females showed a progressively higher incidence in the oldest age quartiles. However, outcomes such as mortality, Intensive Care Unit admission, and length of hospital stay were worse in the older male AKI population.

**Conclusions:**

AKI incidence and in-hospital outcomes differ between males and females in an age-dependent manner. These findings emphasize the relevance of considering sex by age interaction when investigating AKI epidemiology and outcomes.

## Graphical Abstract

**Graphical Abstract:**
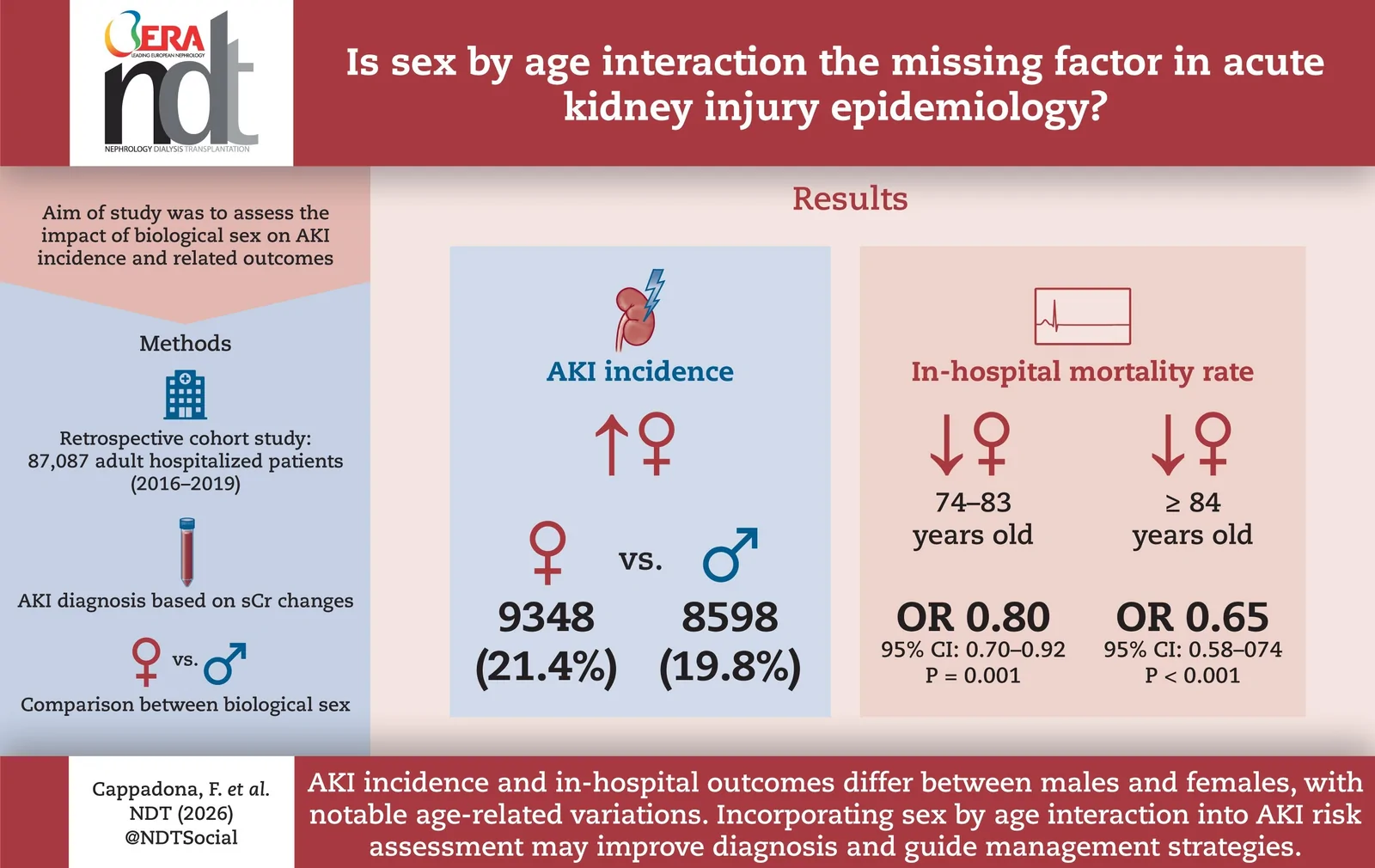

KEY LEARNING POINTS **What was known:**

- AKI is a common and clinically relevant complication in hospitalized patients.
- AKI epidemiology and outcomes are influenced by multiple biological and patient-related factors.
- Conflicting evidence exists regarding the impact of sex and age on AKI epidemiology.

**This study adds:**

- In a large cohort of hospitalized adults, AKI incidence differs between males and females.
- Sex-related differences in AKI incidence vary considerably across age groups.
- Among patients with AKI, sex by age patterns are observed for in-hospital outcomes.

**Potential impact:**

- Analyses based solely on sex comparisons, without accounting for age-dependent variability, may not adequately describe AKI epidemiology.
- Sex by age interaction should be incorporated into AKI definitions and diagnostic frameworks.
- Tailored diagnostic approaches and management strategies should consider sex by age interaction to warrant equitable and individualized kidney care.

## INTRODUCTION

Acute kidney injury (AKI), a complex clinical syndrome characterized by an abrupt decline of kidney excretory function, represents a major health and economic burden worldwide [1]. AKI can be diagnosed and graded according to the Kidney Diseases Improving Global Outcomes (KDIGO) 2012 guidelines [2]. The incidence of AKI varies in relation to the specific clinical setting, ranging from approximately 10% for Community-Acquired AKI to 20%–50% for Hospital-Acquired AKI (HA-AKI) and reaching an incidence over 50% in critically ill patients admitted to Intensive Care Unit (ICU) [3, 4]. Independent of the clinical setting, AKI and its severity definitely correlate with morbidity and mortality [5]. Given the clinical relevance of AKI, the identification of individual patient risk factors for AKI development and outcomes holds a pivotal role for the prevention, identification, and successful treatment of this complex syndrome. Many factors, such as dehydration, comorbidities, and demographic characteristics, influence the risk of developing AKI [6].

Among these, biological sex—defined by biological variables underlain by different sex chromosomes—and age may play a key role; however, their impact on AKI remains unclear and not totally considered. The biological mechanisms involved in sex differences are thought to affect kidney development and possibly the predisposition to develop disease, including AKI [7, 8]. In preclinical models of AKI following ischemia reperfusion injury (IRI), the presence of testosterone is associated with an increased susceptibility to tubular injury that can be modulated by orchiectomy [9, 10]. Likewise, the presence of female hormones is generally found to be protective against IRI, reducing tubular and endothelial cell injury and AKI-associated inflammatory response [11–13]. However, these results are sometimes not consistent, as shown by the reported potential kidney protective effect of testosterone and the evidence that human males undergoing androgen-depriving therapy display a higher risk of AKI [14, 15]. More recently, the role of age and sex differences in experimental models of AKI-to-Chronic Kidney Disease (CKD) transition has also been reported [16]. In humans, male sex seems to be associated with an increased risk of AKI in many subgroups, including HA-AKI, post-surgical AKI, AKI in the ICU, and even a higher risk of developing AKI requiring dialysis [17, 18]. On the other hand, in cardiac surgery-associated AKI, female sex has been reported as a risk factor, at least partially explained by the older age and higher surgical complexity [17, 19]. The evidence that sex- and age-dependent hormonal factors could interplay in the risk of developing AKI is also supported by preclinical evidence [20, 21]. Nevertheless, sex-specific outcomes of AKI are rarely reported in clinical studies, and the clinical importance of this common syndrome in hospitalized patients when stratified by sex or age is largely unknown [22]. For these reasons, this study aimed to investigate the impact of sex by age interaction on AKI epidemiology and outcomes in a large cohort of hospitalized adults.

## MATERIALS AND METHODS

### Study design and population

This study is a part of an epidemiological analysis designed to evaluate AKI incidence and related clinical outcomes in Italian hospitals supported by the AKI & CRRT Project Group of the Italian Society of Nephrology (SIN) [23]. Specifically, we performed a retrospective observational study in the hospitalized population admitted to Policlinico Universitario San Martino, Genova, Italy, and Azienda Ospedaliera Universitaria Maggiore della Carità, Novara, Italy.

Inclusion criteria were (a) adult age (age ≥18 years); (b) first hospital admission from 1 January 2016, to 31 December 2019, and (c) at least two measurements of serum creatinine (sCr).

The only exclusion criteria were the presence of CKD stages 4–5 identified by the ICD-9-CM (International Classification of Disease, 9th Revision, Clinical Modification) diagnosis codes reported on the Hospital Discharge Form (HDF).

The institutional review boards approved the study protocol (Genova: N. Registro CER Liguria: 515/2020; Novara: Protocollo 530/CE, Studio n. CE 220/19 NOV-AKI Study) and waived the need for informed consent. The study was performed in accordance with the Declaration of Helsinki.

### Data collection

All data were extracted from the hospital electronic database. We exported the following demographic, clinical, and laboratory data: age, sex, comorbidities, sCr, ward of admission (emergency medicine, medical, surgical, or ICU), length of hospital stay (LOS), death, and main outcomes. Comorbidities, including heart failure (HF), diabetes, acute myocardial ischemia, CKD, and sepsis, were identified using ICD-9-CM codes. The sCr levels were collected at admission and discharge: the highest and lowest sCr for each patient were also recorded.

### Definitions

The presence and stages of AKI were recorded by dividing the peak sCr by the lowest sCr during hospitalization (peak sCr/lowest sCr), under the assumption that the lowest sCr represented baseline kidney function.

We defined AKI according to the “extended” KDIGO Clinical Practice Guideline, based solely on sCr changes without considering specific time intervals between determinations [2]. We reported each stage according to the KDIGO framework as stages 1, 2, and 3. These correspond to stage 1 (1.5–1.9 times baseline sCr), stage 2 (2–2.9 times baseline sCr), and stage 3 (3 or more times their baseline creatinine or the need for dialysis).

Urinary output was not considered as a criterion for AKI due to the retrospective nature of the study and the limited data collected outside the ICU.

The incidence of AKI was also described by the rate of report on HDF (code 584.5–584.9), and the recognition of AKI was calculated by comparing sCr-based AKI incidence with the incidence of report on HDF [24]. Renal recovery was calculated in AKI patients alive at the time of hospital discharge by dividing the sCr at discharge by the lowest sCr during hospitalization (discharge sCr/lowest sCr). Patients were considered recovered when they did not meet KDIGO criteria for AKI (ratio <1.5) any longer. Conversely, patients with an sCr ratio of 1.5 or higher were classified as having acute kidney disease [25].

The estimated glomerular filtration rate (eGFR) was calculated using the Chronic Kidney Disease Epidemiology Collaboration creatinine-based equation [26].

### Outcomes

The primary outcome of this etiological study is to assess the incidence of in-hospital AKI correlated to sex and age differences. The secondary outcomes are mortality and renal recovery according to sex and age. Moreover, we also considered the LOS, the type of discharge, and kidney function at discharge in AKI patients as secondary outcomes.

### Statistical analysis

Normally distributed variables are presented as mean ± SD and were compared using an independent or paired *t*-test when appropriate. Comparisons between groups were made by analysis of variance. Non-normally distributed variables are presented as median plus interquartile range and were compared using non-parametric tests. Comparisons of proportions were made using the χ^2^-test or Fisher’s exact test when appropriate.

The association between biological sex and the occurrence of AKI during hospitalization was assessed using logistic regression models, with AKI development as the outcome. To explore effect modification by age, analyses were stratified by age quartiles, and sex-specific odds ratios (ORs) with 95% confidence intervals (95% CIs) were estimated within each age group.

Because biological sex and age are fixed characteristics that precede hospitalization and cannot be causally influenced by downstream clinical variables, models evaluating sex and age as exposures were not adjusted for comorbidities, baseline serum creatinine, ICU admission, or other in-hospital variables, which may act as mediators or colliders rather than confounders [27].

Among patients who developed AKI, logistic regression was used to assess the association of sex and age with clinically relevant outcomes (e.g. in-hospital mortality, ICU admission, prolonged length of stay, discharge disposition, renal recovery). For these analyses, the outcome was defined as the occurrence of the specific clinical endpoint during the hospitalization.

Given the lack of information on the exact timing of AKI onset and the absence of post-discharge follow-up data, time-to-event analyses were not performed to avoid potential immortal time and selection bias. Follow-up for all outcome analyses was therefore restricted to the in-hospital period.

Statistical significance was defined as a two-sided *P*-value <.05. All analyses were performed using Stata version 14.2 (StataCorp, College Station, TX, USA).

## RESULTS

### Study population

We collected data from 87 087 patients who fulfilled the inclusion criteria. The average age was 69.2 ± 17.7 years, and 43 467 (49.9%) were male. As reported on the HDF 8455 (9.7%) patients were diabetic (DM), 7767 (8.9%) had HF, and 5924 (6.8%) had CKD. Sepsis occurred in 3361 (3.9%) patients. At the time of admission, mean sCr was 0.9 (0.7–1.2) mg/dl, corresponding to a mean eGFR of 83.56 ml/min/1.73 m^2^ (IC 56–99). Males had higher baseline values of sCr (0.99 mg/dl, CI 0.8–1.3 vs 0.79 mg/dl, CI 0.6–1.01) compared to females.

As reported in Table 1, medical wards represented the main unit of admission (*n* = 37 902, 43.5%), followed by emergency medicine (*n* = 23 467, 27.0%), surgery (*n* = 22 569, 25.9%), and ICU (*n* = 3147, 3.6%).

**Table 1:** Baseline characteristics of patients admitted to the hospital categorized by sex.

	All patients 87 087	Males 43 444	Females 43 604	*P*-value
Age, years	69.2 ± 17.7	68.5 ± 16.5	69.9 ± 18.9	<.0001
Type 2 diabetes mellitus, *n* (%)	8455 (9.7)	4678 (10.8)	3777 (8.7)	<.0001
Heart failure, *n* (%)	7767 (8.9)	3711 (8.5)	4056 (9.3)	<.0001
Chronic kidney disease, *n* (%)	5924 (6.8)	3329 (7.7)	2595 (6)	<.0001
Acute myocardial ischemia, *n* (%)	3267 (3.7)	2008 (4.6)	1259 (2.9)	<.0001
Sepsis, *n* (%)	3361 (3.9)	1748 (4.0)	1613 (3.7)	.013
Serum creatinine at admission, mg/dl	0.9 (0.7–1.2)	0.99 (0.8–1.3)	0.79 (0.6–1.01)	<.0001
eGFR at admission, (ml/min/1.73 m^2^)	83.5 (56–99)	84.2 (57.8–99.0)	82.6 (54.5–99.1)	<.0001
Admission department, *n* (%)				<.0001
- Medical	37 902 (43.5)	20 238 (46.5)	17 664 (40.5)	
- Surgical	22 569 (25.9)	9982 (23.0)	12 587 (28.9)	
- ICU	3147 (3.6)	1960 (4.5)	1187 (2.7)	
- Emergency	23 467 (27.0)	11 286 (26.0)	121 181 (51.9)	
Discharge department, *n* (%)				<.0001
- Medical	53 355 (61.3)	27 801 (63.9)	25 554 (58.6)	
- Surgical	24 883 (28.6)	11 485 (26.4)	13 398 (30.7)	
- ICU	1693 (1.9)	963 (2.2)	730 (1.7)	
- Emergency	7156 (8.2)	3218 (7.4)	3938 (9.1)	

Abbreviations: eGFR: estimated glomerular filtration rate; ICU: intensive care unit.

In comparison to males, females were admitted more frequently to emergency and surgery departments (52% vs 26% and 29% vs 23%, respectively), but less to the ICU (1.7% vs 2.2%) (Table 1).

### AKI incidence and distribution according to sex and age

During a median hospitalization of 7 days (IQR 4–13 days), AKI occurred in 17 946 (20.6%) patients (Table 2). The overall incidence of AKI was significantly higher in females (*n* = 9348, 21.4%) than in males (*n* = 8598, 19.8%, *P* < .0001).

**Table 2:** Clinical characteristics and AKI severity in male and female patients with AKI.

	All AKI	Males	Females	*P* value
Patients, *n* (%)	17 946 (20.6)	8598 (19.8)	9348 (21.4)	<.0001
Age, years	74.8 ± 14.7	72.5 ± 14.6	76.9 ± 14.6	<.001
Comorbidities				
Type 2 diabetes mellitus, *n* (%)	2009 (11.2)	994 (11.6)	1015 (10.9)	.136
Heart failure, *n* (%)	2645 (14.7)	1197 (13.9)	1448 (15.5)	.003
Chronic kidney disease, *n* (%)	1704 (9.5)	880 (10.2)	824 (8.8)	.001
Acute myocardial ischemia, *n* (%)	1014 (5.6)	527 (6.1)	487 (5.2)	.008
Sepsis, *n* (%)	1913 (10.7)	979 (11.4)	934 (10.0)	.002
Kidney function				
Serum creatinine at admission, mg/dl	1.55 ± 1.47	1.73 ± 1.7	1.38 ± 1.36	<.001
eGFR at admission (ml/min/1.73 m^2^)	59.5 (34.4–87.9)	61.7 (35.7–90.0)	57.6 (33.3–86.3)	<.001
AKI KDIGO stages, *n* (%)				.004
Stage 1	10 679 (59.5)	5065 (58.9)	5614 (60.1)	
Stage 2	4611 (25.7)	2181 (25.4)	2430 (26.0)	
Stage 3	2656 (14.8)	1352 (15.7)	1304 (14.0)	
Diagnosed AKI on HDF	3541 (4.1)	1897 (4.4)	1668 (3.8)	<.0001

Abbreviations: eGFR: estimated glomerular filtration rate, AKI: acute kidney injury, KDIGO: Kidney Diseases Improving Global Outcomes, HDF: hospital discharge form.

Compared to the non-AKI population, patients with AKI were significantly older, presented a higher prevalence of CKD (9 vs 6.1% of the non-AKI group, *P* < .001), diabetes (11.2 vs 9.3%, *P* < .001), HF (14.7 vs 7.4%, *P* < .001), and sepsis (10.7 vs 2.1%, *P* < .001). Furthermore, patients experiencing AKI had significantly higher baseline sCr levels (1.55 ± 1.53 vs 1 ± 1 mg/dl, *P* < .001).

Looking at sex-based differences of patients experiencing AKI, females were older than males (76.9 ± 14.6 vs 72.5 ± 14.6 years, *P* < .001) and had a higher HF prevalence (15.5% vs 13.9%), whereas males had more CKD, acute myocardial ischemia, and sepsis (Table 2). Moreover, eGFR at the time of admission was significantly lower in females: 82.6 ml/min/1.73 m^2^ (CI 54.5–99.1) vs 84.2 ml/min/1.73 m^2^ (CI 57.8–99.0), *P* < .001 (Table 2).

Regarding the severity of AKI and its distribution across males and females, stage 1 AKI was the most represented in both sexes (*n* = 10 679, 59.5%), followed by stage 2 AKI (*n* = 461, 25.7%) and stage 3 AKI (*n* = 2656, 14.8%). However, male patients had a significantly higher incidence of stage 3 AKI compared to females (15.7 vs 14%, *P* = .004).

AKI was generally under-recognized in HDF, only in 3541 of 17 946 (4.1%) across the whole study population. However, female patients tended to have an even lower rate of AKI reports than males (3.8 vs 4.4%, *P* < .0001).

When analyzing AKI recognition by severity (Table 3), we observed that recognition rates increased with higher AKI stages. Moreover, across all stages, females were less likely than males to have AKI formally recorded on HDF, although this difference reached statistical significance only for stage 1 AKI (in both the contingency analysis and the logistic model).

**Table 3:** Incidence and logistic models for AKI detection in hospitalized patients developing AKI (diagnosed according to sCr changes).

Stage	Males with AKI	Males with AKI on HDF	Females with AKI	Females with AKI on HDF	*P* value χ²	OR AKI recognition (females vs males)	*P* value logistic regression
1	5065	405 (8%)	5614	330 (5.9%)	<.001	0.72 (0.62–0.83)	<.001
2	2181	342 (15.7%)	2430	342 (14.1%)	.13	0.88 (0.75–1.03)	.125
3	1352	394 (29.1%)	1304	369 (28.3%)	.66	0.96 (0.81–1.13)	.63

Abbreviations: AKI: acute kidney injury, HDF: hospital discharge form.

### AKI risk according to age and sex

We then stratified the population into age quartiles, and the results were as follows:

First from 18 to 59 years, *n* = 22.894

Second from 60 to 73 years *n* = 21.177

Third from 74 to 83 years *n* = 23.241

Fourth 84 years and older (oldest 106) *n* = 19.802

Regarding sex differences across age quartiles, females were slightly more common in the first quartile (females 51%, males 49%, *P* = .0001), while the second and third quartiles showed a male predominance (41% vs 59% and 48% vs 52%, respectively, *P* = .0001). The fourth quartile exhibited the greatest sex distribution disparity, with 61% females and 39% males (*P* = .0001). The overall incidence of AKI increased proportionally with age: first quartile had 2630 cases (11.5%), second quartile 4095 (19.3%), third quartile 5539 (23.9%), and fourth quartile 5682 (28.9%) (*P* < .0001).

Figure 1 shows the data on AKI incidence and severity in both sexes across the different age groups.

**Figure 1:**
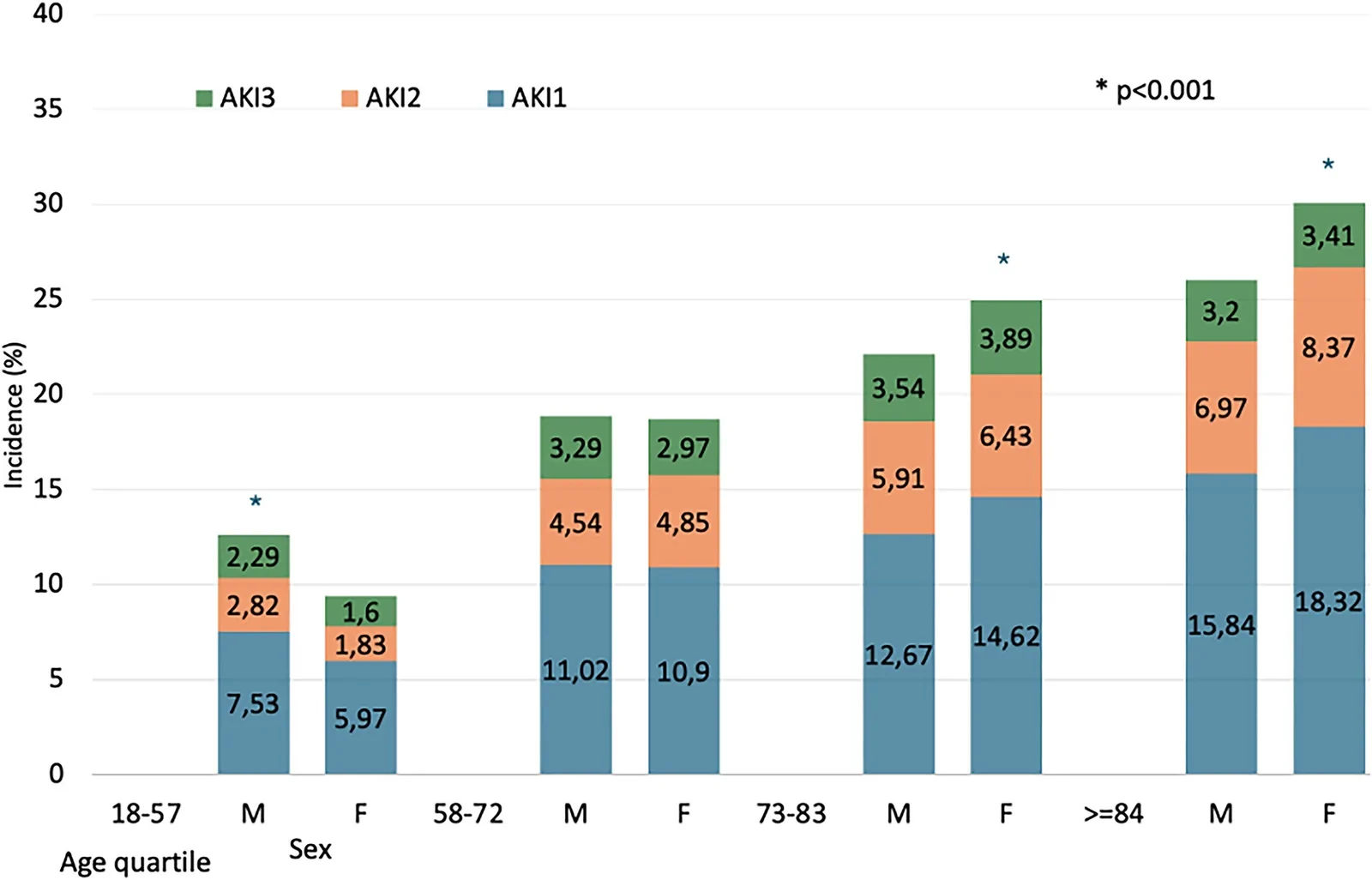
Distribution of AKI and AKI KDIGO stages between males and females across the four age quartiles.

In the first age quartile, AKI was more incident in males (1463, 13.1% vs 1167, 10%, *P* ≤ .001), in the second there was no difference (2390, 19.1% vs 1705, 19.6%, *P* = .373). However, in the higher quartiles, females developed AKI more frequently: 2801, 25.2% vs 2738, 22.6% (*P* ≤ .001) and 3675, 30.4% vs 2007, 26% (*P* ≤ .001).

Logistic regression analysis showed that, while in the first quartile the risk of developing AKI did not correlate with sex, in older groups female sex was increasingly associated with AKI development (second quartile OR 1.03, IC 0.96–1.10, *P* = .38; third quartile OR 1.15, IC 1.08–1.22, *P* < .001; fourth quartile OR 1.24 IC 1.16–1.31, *P* < .001) (Table 4).

**Table 4:** Univariate logistic regression analysis for AKI risk across the four age quartiles, comparing female and male patients.

	Q1			Q2		
	18–59 years			60–73 years		
	OR	95% CI	*P*	OR	95% CI	*P*
	F vs M			F vs M		
Sex (female)	0.72	0.66–0.78	<.001	1.03	0.96–1.10	.38
	Q3			Q4		
	74–83 years			≥84 years		
	OR	95% CI	*P*	OR	95% CI	*P*
	F vs M			F vs M		
Sex (female)	1.15	1.08–1.22	<.001	1.24	1.16–1.31	<.001

Abbreviations: AKI: acute kidney injury; OR: odds ratio.

### Clinical outcomes among patients with AKI across sex and age

As shown in Table 5, in-hospital clinical outcomes among AKI patients, including mortality, ICU admission, and LOS, varied according to sex and age. In contrast, discharge-related outcomes, such as discharge type and renal recovery rates, were similar between males and females across age groups.

**Table 5:** Outcomes in AKI patients based on sex and age quartiles.

AGE quartile	*N*	In-hospital death, *n* (%)	ICU admission, *n* (%)	LOS >15 days, *n* (%)	Protected discharge, *n* (%)	Renal recovery, *n* (%)
Q1 (18–59 y)						
Male	1463	143 (9.8)	357 (24.4)	1834 (16.4)	427 (32.3)	1083 (82)
Female	1167	97 (8.3)	184 (15.8)	1658 (14.2)	227 (21.2)	853 (79.7)
*P*	-	.196	<.0001	<.0001	<.0001	.149
OR F vs M (95%CI)	-	0.84 (0.64–1.09)	0.58 (0.48–0.70)*	0.93 (0.79–1.08)	0.56 (0.47–0.68)	0.86 (0.7–1.06)
Q2 (60–73 y)						
Male	2390	391 (16.4)	392 (16.4)	2281 (18.3)	666 (33.3)	1608 (80.5)
Female	1705	251 (14.7)	259 (15.2)	1861 (24.4)	504 (34.7)	1165 (80.1)
*P*	-	.155	.296	<.0001	.409	.77
OR F vs M (95%CI)	-	0.88 (0.74–1.05)	0.91 (0.377–1.08)	1.10 (0.97–1.24)	1.06 (0.92–1.22)	0.98 (0.82–1.16)
Q3 (74–83 y)						
Male	2738	548 (20.0)	336 (12.3)	2615 (21.6)	835 (38.1)	1800 (82.2)
Female	2801	468 (16.7)	264 (9.4)	2556 (23.0)	920 (39.4)	1902 (81.5)
*P*	-	.001	.001	.014	.368	.56
OR F vs M (95%CI)	-	0.80 (0.70–0.92)*	0.74 (0.63–0.88)*	0.93 (0.83–1.03)	1.05 (0.94–1.19)	0.96 (0.82–1.11)
Q4 (≥84 y)						
Male	2007	548 (27.3)	98 (4.9)	1683 (21.8)	584 (40.0)	1252 (85.9)
Female	3675	726 (19.8)	129 (3.5)	2610 (20.6)	1229 (41.7)	2512 (85.2)
*P*	-	<.001	.012	.654	.295	.56
OR F vs M (95%CI)	-	0.65 (0.58–074)*	0.71 (0.54–0.93)*	0.82 (0.74–0.92)	1.07 (0.94–1.21)	0.95 (0.79–1.13)

Abbreviations: AKI: acute kidney injury; ICU: intensive care unit; LOS: length of stay. * *P* < .05.

With regard to in-hospital mortality, we observed no difference in mortality rates among patients included in the first two age quartiles, while mortality rates were markedly lower in older female patients.

Accordingly, logistic regression analyses stratified by age quartiles showed a significant sex by age interaction for in-hospital mortality among AKI patients, with female sex associated with lower odds of death in the two oldest age groups [third quartile OR 0.80 (95%IC 0.70–0.92), *P* = .001; fourth quartile OR 0.65 (95%IC 0.58–074), *P* < .001].

## DISCUSSION

AKI is a frequent and clinically significant complication in hospitalized patients, profoundly impacting short- as well as long-term outcomes [1, 28]. The pathophysiology of AKI is multifactorial and remains largely unelucidated. In this study, we explored the potential impact of two universal factors: sex and age.

In our large cohort of hospitalized adults, AKI occurred in approximately 20% of patients, with stage 1 predominating across both sexes. Although females experienced a higher overall incidence of AKI, stage 3 AKI was more frequent in males (15.7% vs 14%), confirming findings from a recent UK registry-based study [29]. This observation, combined with the higher ICU admission rate among men, suggests that AKI may present more severely in males. Unfortunately, the lack of detailed ICU data precluded analysis of admission indications and whether ICU admission was related to AKI complications or other causes. Another important finding is the very low AKI reporting rate at hospital discharge (4.1%), consistent with previous studies highlighting under-recognition of AKI in hospitalized patients [30, 31]. This pattern, observed across different populations and AKI stages, appeared to be sex-biased at least for the less severe AKI. Such underdiagnosis may have important implications for post-discharge management, follow-up, and prevention of AKI-to-CKD progression.

Beyond these descriptive results, our data reveal a clear interaction between sex and age in shaping AKI risk and outcomes. Male patients exhibited higher AKI rates in younger age groups (18–73 years), whereas the highest incidence occurred among females aged ≥84 years. This is a key finding, given the ongoing debate regarding how patient-specific factors, such as genetic predisposition, age, and sex, modulate AKI pathogenesis and influence its epidemiology and clinical trajectory.

Aging kidneys undergo progressive structural and functional changes, including diminished excretory capacity and altered cellular responses to injury [32, 33]. Conversely, sex-related biological differences arise from complex molecular pathways involving hormonal regulation of water balance and tubular transport (e.g. sodium–glucose cotransporters), as well as distinct immunological and hemodynamic profiles [34, 35]. For instance, middle-aged and older men demonstrate enhanced vasoconstriction through upregulation of type A endothelin receptors, whereas premenopausal women exhibit endothelin-mediated vasodilation predominantly through type B receptors [36]. Furthermore, women also generally have lower aldosterone levels, and estrogens downregulate angiotensin-converting enzyme and the angiotensin II type 1 receptor [37]. These sex-specific differences likely influence AKI susceptibility and progression to CKD. However, although experimental studies consistently show greater AKI vulnerability and accelerated CKD progression in male rodents, human data remain less consistent [8, 12, 15, 20].

Early clinical guidelines, including KDIGO 2012, suggested that female sex correlates with higher rates of HA-AKI; however, larger epidemiological studies have not reliably confirmed these findings [38, 39]. Moreover, evidence of sex-based disparities in AKI incidence and short-term outcomes is still unsatisfying and reports contrasting data. In this view, a recent secondary analysis of the STARRT-AKI, a randomized clinical trial in critically ill adults, found no modification by sex on the relationship between timing of kidney replacement therapy (KRT) initiation, 90-day mortality, KRT dependence, or healthcare resource use [40].

Within this complex and sometimes contradictory landscape, our large cohort study offers novel insights, showing that the highest AKI incidence observed among females was largely driven by the oldest age groups. Moreover, in the last two age quartiles, female sex was associated with a higher risk of AKI. These findings suggest that the risk of developing AKI can be dependent on sex but that sex relationship with AKI incidence is not continuous with age, showing a conversion between the third and fourth quartiles (74–84 years) and leading to the observation that male patients are at higher risk in the younger population, whereas female patients are at higher risk in the older cohort.

These findings should be interpreted cautiously and viewed as descriptive associations rather than causal effects. Indeed, the observed pattern may reflect a survivor bias or unique pathophysiological characteristics of AKI in very old females that are potentially related to transient, hemodynamic insults rather than a sustained and prolonged injury.

Moreover, comorbidities may misperceive the association between sex and AKI-related mortality, as recently reported for male patients [41].

An alternative plausible explanation for these discrepancies is that standard serum creatinine–based AKI definitions, as similarly discussed for CKD, may perform poorly in older adults, particularly females with reduced muscle mass or sarcopenia [42, 43].

Complementary diagnostic tools such as cystatin C or urinary biomarkers warrant further investigation and validation to establish more accurate and clinically relevant definitions of AKI in this specific population [25]. However, even in this case, caution is needed, as recent studies suggest that biomarker performance may differ by sex, reinforcing the need for sex-specific approaches in AKI research and management [44]. Anyway, previous cohort studies suggested that the relationship between age, mortality, and AKI risk in older adults is not strictly age dependent. Xu *et al*. reported that advanced age did not show a linear correlation with increased AKI risk or mortality in elderly patients [45]. Similarly, Medunjanin *et al*. observed that advanced age was associated with a lower incidence of KRT following AKI [46].

Our study has several strengths, including the large sample size and consistent design, but also some important limitations.

In particular, although our results are consistent with those reported in large epidemiological studies, the pragmatic approach used to define AKI, while reflective of real-world clinical practices, introduces a potential source of variability. Specifically, the choice of baseline serum creatinine, lack of specific time points to determine sCr, and the absence of data on urine output may significantly impact the accuracy of AKI incidence assessments and recognition [47].

The absence of urine output data is particularly relevant among older patients, in whom serum creatinine may be an unreliable diagnostic marker due to malnutrition, sarcopenia, and physical inactivity. This limitation, however, is common to much of the current AKI epidemiological literature [48, 49].

Collectively, these issues raise concerns about the validity of a creatinine-only AKI definition, especially in an aging population, and support the need for future multidimensional diagnostic approaches that integrate kidney injury biomarkers and functional measures (e.g. renal functional reserve), as also emphasized by recent KDIGO initiatives [50].

An additional limitation is the lack of information on the exact timing of AKI onset, precluding time-to-event analyses. However, because AKI was identified retrospectively based on serum creatinine changes occurring at any time during hospitalization, analyses restricted to patients who developed AKI may still be susceptible to immortal time and selection bias. Although we limited outcome analyses to in-hospital binary events and avoided time-to-event approaches to minimize this issue, residual bias cannot be fully excluded.

Moreover, we have no data on causes of hospitalization, and potential selection bias cannot be excluded, as severe AKI might represent both a cause and a consequence of clinical deterioration. Comorbidity data were derived solely from administrative records, potentially leading to misclassification. Finally, while the age-quartile stratification allowed robust statistical analysis, it may not fully reflect physiological heterogeneity. In particular, because the cohort was overall elderly, the first quartile was broad (18–59 years), whereas the third quartile was relatively narrow (74–83 years), potentially limiting the generalizability and emphasizing the need for population-specific epidemiological frameworks [51]. Nevertheless, our data clearly indicate that the incidence, severity, and outcomes of AKI differ between males and females across the lifespan, reflecting complex interactions between biological and clinical factors. Consequently, adopting a sex–age–specific perspective in AKI research may help to better describe AKI epidemiology and short-term outcomes and to inform the evaluation of long-term sequelae. Indeed, although long-term outcomes were not assessed in the present study, previous cohort studies have identified female sex as an independent risk factor for AKI-to-CKD progression [52]. Incorporating sex by age interaction into epidemiological and clinical studies may improve AKI recognition, enable more personalized treatment strategies, and enhance prevention of long-term renal complications [53]. Appropriately designed studies should further explore the prognostic implications of these findings. Ultimately, translating these insights into clinical practice will be critical for achieving equitable and personalized kidney care.

## Data Availability

The datasets used and/or analyzed during the current study are available from the corresponding author on reasonable request.
